# Topical sirolimus 0.1% for treating cutaneous microcystic lymphatic malformations in children and adults (TOPICAL): protocol for a multicenter phase 2, within-person, randomized, double-blind, vehicle-controlled clinical trial

**DOI:** 10.1186/s13063-019-3767-8

**Published:** 2019-12-17

**Authors:** Sophie Leducq, Agnès Caille, Sébastien Barbarot, Nathalie Bénéton, Didier Bessis, Olivia Boccara, Anne-Claire Bursztejn, Christine Chiaverini, Anne Dompmartin, Catherine Droitcourt, Valérie Gissot, Dominique Goga, Laurent Guibaud, Denis Herbreteau, Anne Le Touze, Christine Léauté-Labrèze, Gérard Lorette, Stéphanie Mallet, Ludovic Martin, Juliette Mazereeuw-Hautier, Alice Phan, Patrice Plantin, Isabelle Quéré, Pierre Vabres, Hélène Bourgoin, Bruno Giraudeau, Annabel Maruani, Groupe de Recherche de la Société Française de Dermatologie Pédiatrique

**Affiliations:** 1 INSERM U1246 -SPHERE « MethodS in Patients-centered outcomes and HEalth REsearch », University of Nantes, University of Tours, 37000 Tours, France; 2 Department of Dermatology and Reference Center for Rare Diseases and Vascular Malformations (MAGEC), CHRU Tours, Avenue de la République, 37044 Tours, Cedex 9 France; 3 Clinical Investigation Center, INSERM 1415, CHRU Tours, 37000 Tours, France; 4 Department of Dermatology, University Hospital Center of Nantes, 44000 Nantes, France; 5 Department of Dermatology, Hospital Center of le Mans, 72037 le Mans, France; 6 Department of Dermatology, University Hospital Center of Montpellier, 34000 Montpellier, France; 7 Department of Dermatology and Reference center for genodermatoses and rare skin diseases (MAGEC), University Hospital Necker-Enfants Malades, 75015 Paris, France; 8 Department of Dermatology, University Hospital Center of Nancy, 54000 Nancy, France; 9 Department of Dermatology, University Hospital Center of Nice, 06000 Nice, France; 10 Department of Dermatology, University Hospital Center of Caen, 54000 Caen, France; 11 Department of Dermatology, University Hospital Center of Rennes, 35000 Rennes, France; 12 Department of Maxillo-Facial surgery, CHRU Tours, 37044 Tours, Cedex 9 France; 13 University Hospital Center of Lyon, Consultation Multidisciplinaire Lyonnaise des Angiomes, 69229 Lyon, Cedex 2 France; 14 Department of Neuroradiology, CHRU Tours, 37000 Tours, France; 15 Department of Pediatric Surgery, CHRU Tours, 37000 Tours, France; 16 Department of Dematology, Pellegrin Children’s Hospital, 33076 Bordeaux, France; 17 Department of Dermatology, University Hospital Center of Marseille, 13885 Marseille, Cedex 5 France; 18 Department of Dermatology, University Hospital Center of Angers, 49000 Angers, France; 19 Reference center for rare skin diseases, Department of Dermatology, University Hospital Center of Toulouse, Paul Sabatier University, 31059 Toulouse, France; 20 Department of Dermatology, University Hospital Center of Lyon, 69229 Lyon, Cedex 2 France; 21 Department of Dermatology, Hospital Center of Quimper, 29107 Quimper, France; 22 Departement of Vascular Medicine, National Reference Centre for Rare Vascular Diseases, EA 2992 Research Team, University of Montpellier, University Hospital Center of Montpellier, 34000 Montpellier, France; 23 Department of Dermatology, University Hospital Center of Dijon, 21000 Dijon, France; 24 Department of Pharmacy, University Hospital Center of Tours, 37000 Tours, France

**Keywords:** Cutaneous microcystic lymphatic malformation, Lymphangiectasia, Sirolimus, Rapamycin, Topical rapamycin, Mammalian target of rapamycin inhibitor, Vascular malformation

## Abstract

**Background:**

Cutaneous microcystic lymphatic malformations (CMLMs) are rare conditions in children and adults. They present as clusters of vesicles full of lymph and blood to various extents, inducing maceration, esthetic impairment, pain, and impaired quality of life. The treatment is challenging. Sirolimus is an inhibitor of mammalian target of rapamycin (mTOR) involved in angio-lymphangiogenesis. Topical sirolimus has recently been reported as effective in a few reports of patients with CMLMs. The objective is to compare the efficacy and safety of a 12-week application of 0.1% topical sirolimus versus topical vehicle in CMLMs in children and adults.

**Methods:**

This French blinded multicenter within-person randomized controlled phase 2 trial aims to include 55 patients aged ≥ 6 years who have a primary CMLM. The CMLM will be divided into two equal areas that will be randomly allocated to 0.1% topical sirolimus or topical vehicle applied for 12 weeks. At the end of the 12-week period, the patient/parent will treat the whole area of CMLM with 0.1% topical sirolimus on remaining lesions, for eight more weeks. Patients will be seen at week 20 (treatment will be stopped) and at month 12 to evaluate long-term efficacy. The primary outcome will be improvement of the CMLM in the area treated with topical sirolimus compared to the area treated with topical vehicle by the investigator physician (blinded to the treatment) with the Physician Global Assessment score at week 12. Secondary outcomes will include: assessment of efficacy by independent experts on the basis of standardized photographs; impact on quality of life; efficacy for oozing, bleeding, erythema, and thickness evaluated by the investigators; and global efficacy as well as efficacy for functional and aesthetic impairment evaluated by the patient. Systemic passage of sirolimus will be measured at weeks 6, 12, and 20, and at week 16 for CMLMs ≥ 900 cm^2^.

**Discussion:**

For patients with CMLMs, topical sirolimus could be a non-invasive and well-tolerated therapeutic option. If the trial demonstrates efficacy and safety of this treatment, this result will lead to a real change in the management of this condition, and 0.1% sirolimus cream would become the first-line treatment.

**Trial registration:**

ClinicalTrials.gov, NCT03972592. Registered on 3 June 2019. EU Clinical Trials Register EudraCT, 2018–001359-11.

## Background

### Background and rationale

Vascular malformations (VMs) are congenital anomalies that can involve four types of vessels (capillary, lymphatic, venous, and arterial vessels) according to their classification as low- or high-flow VMs. The International Society for the Study of Vascular Anomalies updated their classification in 2014 [1]. Among VMs, cystic lymphatic malformations are rare conditions in children and adults (estimated prevalence < 0.1%) [2, 3] consisting of low-flow congenital VMs resulting from abnormal embryologic development of lymphatic vessels [4]. They might be macrocystic, microcystic, or combined and can affect viscera, soft tissues, and/or skin.

Cutaneous microcystic lymphatic malformations (CMLMs), also called lymphangiectasia, present as clusters of vesicles full of lymph and blood to various extents, usually located in a segmental area (head/neck, lower limbs, gluteal area) [5]. They ooze and bleed, inducing maceration, esthetic impairment, scars, pain, bacterial infections, impaired quality of life, and sometimes anemia. The natural history is progressive worsening during life (increase in lesions and complications). There are no guidelines for managing CMLMs, but therapeutic options are sclerotherapy (an efficient treatment for macrocystic lymphatic malformations but disappointing in CMLMs), physical treatments (pulsed-dye laser, CO_2_ laser, radiofrequency, etc.) [6, 7], and surgery [8, 9], but these options are painful and induce inflammation and scars, and their efficacy is usually incomplete and transitory, with the rate of recurrence being very high [10]. Management requires multidisciplinary care. The “wait-and-see” attitude is frequently chosen.

Sirolimus belongs to mammalian target of rapamycin (mTOR) inhibitors. mTOR is a serine/threonine kinase, regulated by phosphoinositide-3-kinase, that acts as a master switch in cell proliferation, apoptosis, metabolism, and angio/lymphangiogenesis. Sirolimus directly inhibits the mTOR pathway, which thereby inhibits cell proliferation, angiogenesis, and lymphangiogenesis [11, 12]. Oral sirolimus is commonly used to prevent rejection of kidney transplants (U.S. Food and Drug Administration [FDA]-approved in this indication) [13]. It was recently reported as an efficient drug to reduce the volume and limit complications in VMs, especially with lymphatic components, in children of all ages [14]. Two randomized controlled trials (RCTs) of oral sirolimus in VMs are ongoing in France and in the United States (ClinicalTrials.gov NCT02509468 and NCT02110069). For CMLMs without underlying painful involvement, many physicians consider oral sirolimus as a too-aggressive option, despite the high level of impairment linked to the condition.

Topical sirolimus is known to be efficient and well tolerated in cutaneous angiofibromas linked to tuberous sclerosis complex: several RCTs have been performed and the drug is currently used in general practice [15, 16]. Recently, topical sirolimus was reported as effective in CMLMs, decreasing the number of vesicles, bleeding, oozing, and pain, in one retrospective study involving 11 patients and in three case reports [17–20]. In the reported cases of CMLMs and angiofibromas, only local side effects were reported, mostly irritative. Systemic passage of sirolimus was not, or almost not, detectable, but the concentration of sirolimus in the preparations and the surface areas that received topical sirolimus were heterogeneous.

### Objectives

We aim to perform a clinical trial (TOPical sIrolimus in CutAneous Lymphatic malformation; TOPICAL) to assess the efficacy and safety of a 12-week application of 0.1% topical sirolimus versus topical vehicle for CMLMs in children and adults.

### Trial design

**Fig. 1:**
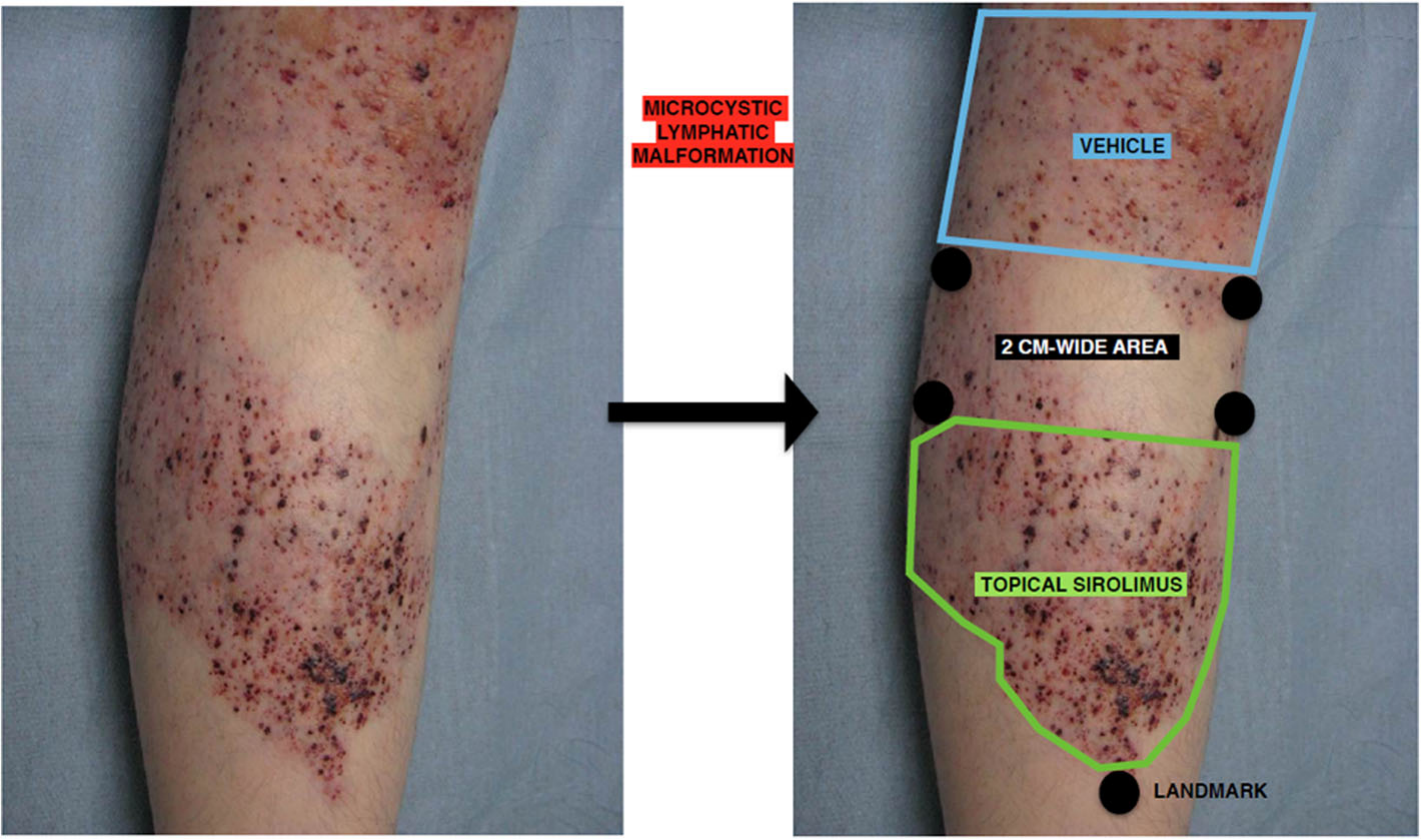
Example of a CMLM of the leg: the investigator will define two areas of similar size and severity by using a ruler, with a separation area of at least 2 cm between both areas (5 points will be drawn on the patient to define the two areas to apply the product in a reproducible way by the nurse and to avoid inter-area contamination). CMLM cutaneous microcystic lymphatic malformation

TOPICAL is a within-person randomized, vehicle-controlled, investigator- and patient-blinded, multicenter, superiority study comparing 0.1% topical sirolimus and topical vehicle treatment for CMLMs. For each patient, the investigator will divide the CMLM area into two equal areas of homogeneous severity separated by a 2-cm–wide strip (Fig. 1). Each area will be randomly allocated to receive 0.1% topical sirolimus or topical vehicle, applied by a nurse, once daily for 12 weeks.

**Fig. 2:**
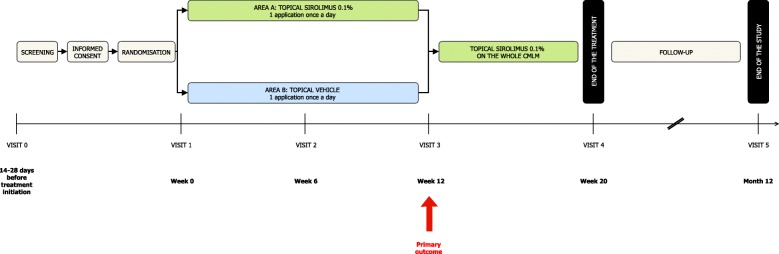
*Flow chart* of the study procedure. CMLM cutaneous microcystic lymphatic malformation

At the end of the 12-week period, the patient/parent will treat the whole area of CMLM with 0.1% topical sirolimus on the remaining lesions, for eight more weeks (Fig. 2). The purpose of this extension phase is to strengthen patients’ adherence to the protocol. Indeed, due to the design, each patient will be his/her own control. Patients may observe an improvement in one of the two areas during the 12-week period and may therefore be attempted to apply the treatment allocated to this area to the whole CMLM, which would introduce group contamination. Therefore, we planned to have nurses apply the treatments; also, patients will be allowed to treat the whole CMLM area with the active drug at the end of the 12-week period. In doing so, we expect patients to be compliant with the protocol and not stop the study.

After week 20 (W20), treatments will be left to the discretion of the investigator.

The within-person design allows for reducing the number of patients to be included; indeed, the population is too rare for a parallel-group trial. Furthermore, the location and severity of CMLMs are heterogeneous among patients and a within-person design has the advantage of reducing inter-observation variability because each patient is his/her own control. Finally, all patients will receive the experimental treatment, which would not be the case with a parallel-group trial.

### Methods: participants, interventions, and outcomes

#### Study setting

The study will involve 16 French tertiary-care hospital centers, all involved in managing vascular anomalies.

#### Eligibility criteria

##### Inclusion criteria

Eligible patients will be aged ≥ 6 years (for a minimal size) and have a diagnosis of primary CMLM confirmed by histopathological or desmoscopic examination [21, 22], with or without an underlying malformation or a syndromic malformation (e.g. Proteus syndrome) responsible for impairment (oozing, bleeding, and/or pain).

We chose to include adults and children aged ≥ 6 years because CMLMs can be seen in both children and adults; however, because the natural history of these malformations is a progressive worsening, impairments of CMLMs are usually not yet major in very young children. Moreover, we need a CMLM area large enough to apply both preparations, with a 2-cm central area in-between, and consider that the required minimum area is 20 cm^2^, which is usually not possible in children aged < 6 years. A 2-cm–wide space will delimit the two areas of the CMLM to avoid contamination between these two areas.

##### Exclusion criteria

Patients with a lymphatic malformation requiring continued background therapy (involving deep organs) will be excluded, as will patients with secondary lymphatic malformations (lymphangiectasia post-radiotherapy, etc.); immunosuppression; ongoing neoplasia; active chronic infectious disease such as chronic viral hepatitis, tuberculosis or HIV infection; and pregnant women and women of childbearing age not using birth control.

We will exclude patients who previously received systemic or topical mTOR inhibitors within 12 months before inclusion or oral or topical steroids within 10 days before inclusion. Finally, we will exclude patients with contraindications to topical sirolimus, such as skin necrosis, local fungal infections, viral infections (e.g. herpes), or bacterial infection (impetigo, etc.) on the site of the CMLM and known allergy to one of the components of the topical sirolimus preparations or the control.

### Intervention

For each patient, the investigator will divide the CMLM area into two equal areas separated by a 2-cm–wide area. Severity of CMLM is usually quite homogeneous; the two areas chosen by the investigator will need to be of similar severity. In case of horizontal malformation, area A will correspond to the area on the left-hand side of the malformation and area B on the right-hand side. In case of vertical malformation, area A will correspond to the area on the upper part of the malformation and area B on the lower part. Each area will be randomly allocated to receive 0.1% topical sirolimus or topical vehicle, applied once daily for 12 weeks (Fig. 1). The 0.1% topical sirolimus application will be prepared and packaged by the pharmacy of the Hospital University Center (CHU) Angers. The formulation of topical sirolimus was developed by the pharmacy team of CHU Angers. Its stability and conservation have been tested and published [23]. Sirolimus cream will be prepared by first solubilizing rapamycin in Transcutol®. Then, the mixture will be progressively added to Excipial® hydrocream. The formulation is 0.03 g rapamycin, 1.5 g Transcutol®, and QS 30 g Excipial® hydrocream, corresponding to a 0.1% concentration. The cream will be packaged in 30-mL aluminum tubes. The stability of this formulation was studied by high-performance liquid chromatography: the rapamycin concentration remained > 95% of the initial concentration for at least 85 days. The odor, appearance, and color of the preparation remained unchanged during storage.

During the double-blind 12-week period, a nurse, trained to the protocol, will apply both topical treatments to avoid inter-group contamination and for better compliance. In addition, treatments will be kept by the nurses.

A research nurse of CHRU Tours will coordinate and support the nurses at home (two nurses will be trained for each patient). A nursing notebook with a guide (explanation for application) and material will be provided to the nurses.

Each of the two products will be applied using a different glove and massaged until complete penetration of thin thickness. The amount to be applied will be defined by using the fingertip unit (FTU), which is widely used in dermatology [24]. One FTU is equivalent to 20–25 mm of cream squeezed onto the fingertip. One FTU is 0.5 g cream and is sufficient to treat an area of skin twice the size of an adult’s hand (including palm and fingers). Thus, the amount of cream to be applied will be adjusted by using the FTU depending on the size of the area. The maximal amount of products to be applied will be 2 g per application for each area (during W0–W12) and 4 g per application for the whole malformation (during W12–W20). Indeed, the maximal size of the malformation expected is 2500 cm^2^ (50 × 50 cm).

Regarding concomitant treatments, bandages in case of oozing and bleeding and topical or systemic antibiotics in case of local bacterial infection will be authorized. Topical steroids, topical immunosuppressive drugs, sclerotherapy, lasers, and surgery on the area will be prohibited. Systemic steroids for a > 3 days, systemic immunosuppressive drugs, and oral mTOR inhibitors will also be prohibited.

A participant may stop study treatment at any time if the participant, investigator, or sponsor feels that it is not in the participant’s best interest to continue. If a participant is withdrawn from treatment because of an adverse effect, the participant will be followed and treated by the investigator until the abnormal parameter or symptom has resolved or stabilized. If a woman becomes pregnant during the clinical trial, treatment will be stopped but all assessments planned in the study will be performed. The investigator must follow the patient until the end of the pregnancy or until its interruption. In case of skin necrosis, which would be unexpected, temporary discontinuation of the treatment is recommended until the next visit. The investigator will check that the skin necrosis is healed.

## Outcomes

### Primary outcome

The primary outcome will be evaluation of the CMLM in the area treated with the intervention (0.1% topical sirolimus) compared to the area treated with topical vehicle (inactive comparator) by the investigator physician (blinded to treatment) by using the 6-point Physician Global Assessment (PGA) score at W12.

There are no specific scores for VMs. We chose the 6-point PGA score because it is relevant for this condition and is easy to understand. The PGA score ranges from 0 (clear) to 5 (severe) and is often used in trials investigating dermatological conditions such as psoriasis [25, 26]. We consider that a 1-point improvement in PGA score is clinically relevant for CMLM.

### Secondary outcomes

Secondary outcomes are clinical assessments of the efficacy of sirolimus as well as safety. Clinical efficacy will be assessed by different endpoints (Table 1).

- Two blinded independent experts will qualitatively assess digital photographs. Experts will be provided photographs for each patient at baseline and at W12; they will be asked to identify, at the end of the study, which area among the two areas received the active treatment;
- Assessment by the physician of the global efficacy of topical sirolimus in the whole CMLM by using the PGA score at baseline, W6, W20, and month 12 (M12);
- Quality of life will be self-assessed by the patient, at baseline, W20, and M12 in comparison to baseline by using the validated Dermatology Life Quality Index (DLQI) or Child-DLQI for children;
- Pain linked to the CMLM will be self-assessed by the patient (and parents for children aged < 16 years) with a visual analog scale (VAS; 0–10) at baseline, W20, and M12;
- Improvement in terms of oozing, bleeding, erythema, and thickness will be assessed by the investigator with blinding to treatment on a VAS scale of 0–10 (0 = no improvement, 10 = recovery) at baseline (on both areas), W12 (on both areas), W20 (on the whole area), and M12 (on the whole area);
- Global improvement in CMLM will be self-assessed by the patient on a VAS scale of 0–10 (and parents for children aged < 16 years) at W12 (in both areas), W20 (on the whole area), and M12 (on the whole area);
- Functional and aesthetic impairment will be self-assessed by the patient (and parents for children aged < 16 years) on a VAS scale of 0–10 at baseline, W20, and M12.

**Table 1:** Schedule of enrolment, interventions, and assessments

	Study period					
	Screening	Inclusion	Follow-up			
	V0	D1 (V1)	W6 (V2)	W12 (V3)	W20 (V4)	M12 (V5)
Timepoint	14–28 days before treatment initiation	–	±3 days	±3 days	±3 days	±5 days
Drug administration		Once daily				
Enrollment						
Eligibility screening	X					
Informed consent	X					
Control of inclusion/exclusion criteria	X					
Randomization		X				
Physical examination		X	X	X	X	X
Height and weight measurement		X				
Vital signs (cardiac frequency, arterial pressure)		X	X	X	X	X
Photographs		X		X		
Location and measure of the CMLM	X					
Definition of the two areas		X				
Urinary pregnancy test for women^a^	X	X	Once a month until S20		X	
Blood sample for tolerance^b^		X		X	X	
Serum level of sirolimus^c^			X	X	X	
Cutaneous effects			X	X	X	
Adverse events			X	X	X	
Delivery of nursing notebook		X	X	X		
Interventions^d^						
Delivery of topical sirolimus		X	X	X		
Delivery control		X	X			
Treatment returns			X	X	X	
Assessments						
PGA score		X	X	X	X	X
Dermatological quality of life scale – DLQI		X			X	X
VAS for self-assessment of pain		X			X	X
VAS for assessment of efficacy by the investigator on oozing, bleeding, erythema, thickness		X		X	X	X
VAS for self-assessment of global efficacy				X	X	X
VAS for self-assessment of functional and esthetic impairments		X			X	X

*CMLM* cutaneous microcystic lymphatic malformation, *PGA* Physician Global Assessment, *VAS* visual analog scale

^a^ If detectable sirolimus level: monthly pregnancy test for three additional months

^b^ Blood count, ionogram, liver function (ASAT, ALAT, gamma-GT), renal function (creatinine), lipids (cholesterol, triglycerides), glycemia. Local analysis

^c^ With CMLM ≥ 30 × 30 cm and/or ≥ 900 cm^2^, a blood sample for evaluation of systemic passage of sirolimus (residual serum level of sirolimus) will be performed at W16

^d^ Application of both topical preparations (intervention or inactive comparator) by a nurse

### Safety

Local adverse events (AEs) in both areas treated with topical sirolimus and topical vehicle before W12 and the whole CMLM at W20 will be recorded. The expected local AEs are mostly irritative (erythema, burning, dryness, itching, pruritus). Systemic passage of sirolimus will be assessed at W6, W12, and W20 (dosage of serum level of sirolimus) and at W16 with CMLM ≥ 30 × 30 cm or ≥ 900 cm^2^. Evaluation of biological safety will be assessed at W12 and W20 in comparison to baseline (we will use biological measurements that are required for assessing safety of oral sirolimus: blood cell count; liver and renal functions; ionography; lipids [cholesterol and triglycerides]; and glycemia).

Classical AEs of oral sirolimus are clinical (mostly mucositis, gastrointestinal effects, fatigue, headaches, hypertension) and biological (thrombocytopenia, leucopenia, anemia, hyperlipidemia, hyperglycemia, hypokalemia, and increased levels of liver enzymes). However, no general AE has ever been reported with topical sirolimus, but several local AEs were reported. Indeed, the risk of a systemic adverse reaction seems low with topical sirolimus, because systemic absorption is very low or hardly detectable. This risk will be assessed with blood samples to assess the safety of the treatment and systemic passage. If a level of sirolimus is detectable, the study treatment will not be stopped. With a level of sirolimus ≥ 15 ng/mL [27], the treatment will be stopped.

Safety evaluation parameters will be performed at each visit by asking the patients/parents to report clinical AEs.

At the screening visit, the inclusion visit, W12, W20, and once a month, a urinary pregnancy test will be performed for women and girls of childbearing potential. With a detectable level of sirolimus, a monthly urinary pregnancy test will be performed for an additional three months.

### Participant timeline

Duration of participation will be 12 months for each patient. The time schedule of enrolment and visits is shown in Table 1.

### Sample size

The study is planned as a within-person design, which means that data from the two areas on a patient will be matched. In addition, the PGA score is an ordered score ranging from 0 (clear) to 5 (severe). Therefore, to estimate the sample size, we used an approach based on continuous data and a paired sample *t* test, assuming that the PGA score difference (between the two areas) follows a normal distribution. Hypothesizing a 1-point difference and a 2.5-point standard deviation would lead to a 0.4 effect size. Assuming a power of 80%, a two-sided type I error rate of 5%, we will need to recruit 52 patients. We plan to recruit 55 patients.

### Recruitment

The recruitment of children with these malformations is facilitated by the fact that all co-investigators belong to tertiary-care centers for vascular anomalies and participate in multidisciplinary consultations on lymphatic malformations. Most co-investigators are already involved in the French trial on oral sirolimus PERFORMUS (NCT02509468) for which recruitment ended in March 2018. Some belong to the French network for research on pediatric dermatology (Groupe de Recherche de la Société Française de Dermatologie Pédiatrique).

### Methods: assignment of interventions

#### Allocation

##### Sequence generation and allocation concealment mechanism

In this within-person trial, areas of the CMLM (area A and area B) will be randomly assigned to the control or experimental group with a 1:1 ratio allocation as per a computer (SAS-based)–generated randomization schedule. Participants will be randomized by using Ennov Clinical©, an online central randomization procedure via the electronic case report form (e-CRF). To ensure allocation concealment, the randomization procedure will not be possible until the participant has been recruited into the trial; in particular, the consent and all eligibility criteria must be collected and met.

##### Implementation

The allocation sequence will be generated by a statistician not involved in the recruitment or follow-up of participants.

### Blinding

Patients, parents, nurses, and investigators will be blinded to the treatment allocated to each area of the CMLM during the first step of the study (until W12, when the primary endpoint will be assessed). To ensure double-blinding, both areas will be randomized and the topical treatments (sirolimus and control) to be applied will have similar packaging. The appearance of the drugs is similar; thus, the active drug (topical sirolimus) and the control cannot be distinguished at drug allocation. In addition, the consistency of the creams is similar.

Topical sirolimus might induce burning or pruritus. However, we do not consider that this side effect would compromise the blinding by allowing the patient to know which area is being treated with the active cream and the control because: (1) identifying precisely which area is itching or burning on a CMLM is difficult; (2) itching and burning might be linked to the CMLM itself; and (3) usually, no inflammation is objectively detectable. The allocation table will be kept in an envelope and will be stored in a secure place. Unblinding will be requested for any reason considered essential by the investigating physician following the procedure determined in advance.

### Methods: data collection, management, and analysis

#### Data collection methods

Table 1 shows data collection according to inclusion and follow-up visits. An individual may be discontinued from study treatment at any time if the individual, the investigator, or the sponsor feels that it is not in the participant’s best interest to continue. If a participant is withdrawn from treatment due to an adverse effect, the participant will be followed and treated by the investigator until the abnormal parameter or symptom has resolved or stabilized. All individuals who discontinue study treatment should be encouraged to complete all remaining scheduled visits and procedures. Once a participant is randomized in the study, every reasonable effort will be made to follow the participant for the entire study period even if there is a deviation from the intervention protocol, an early discontinuation of study treatment, or one missed follow-up visit. If an individual is lost to follow-up, every possible effort must be made by the study center to contact the individual and determine the reason. The measures taken to follow-up must be documented. Primary and secondary outcomes, as well as safety, will be collected for all participants even for participants who discontinue or deviate from intervention protocol.

### Data management

An e-CRF will be developed by using Ennov Clinical© software. The e-CRF will be managed in agreement with INSERM CIC 1415 Standardized Operating Procedures (SOPs). Data from investigating centers will be entered using a secure website monitored by clinical research associates, and queries will be edited by data managers, in agreement with an *a priori*-specified data-management plan. A blinded review will be performed before locking the database. The database will be locked in agreement with INSERM CIC 1415 SOPs and data will be extracted in a SAS format or another format, according to statistical requirements. Raw data will be stored in an XML format.

### Statistical methods

The statistical analysis will be conducted according to the intention-to-treat (ITT) principle: areas will be analyzed within the groups to which they were initially randomized even if the patient do not adhere to the intervention. We will use multiple imputation to account for missing variables. Considering the primary outcome, the study is planned as a within-person trial with the specificity that in these types of studies, each patient serves as his/her own control. In this way, we will have a paired sample. For the primary analysis, the PGA score at W12 will be compared between the two areas by paired Student’s *t* test. For secondary outcomes, PGA score, physician assessment of efficacy, and patient self-assessment will be analyzed by the same approach (i.e. paired Student’s *t* test). Considering efficacy seen on digital photographs (i.e. correct identification of intervention/control-treated area), interpretation by dermatologic experts will be considered correct or false, and the proportion of correct interpretation will be estimated and provided with 95% confidence intervals. The proportion of correct interpretation will be compared to the theoretical 50% value, corresponding to a random assessment. At W20 and M12, PGA score, physician assessment of efficacy, and patient self-assessment will be reported using descriptive statistics. Change in quality of life and in pain between baseline and M12 will be analyzed by using a linear mixed effects model with a random intercept to account for repeated measures for the same patient. Regarding AEs and systemic passage of sirolimus, descriptive statistics (numbers and percentages) will be calculated. No subgroup analyses or adjusted analyses are planned.

### Methods: monitoring

#### Data monitoring

A clinical research assistant will be responsible for coordinating the study: the assistant will be responsible for the logistics and monitoring the study, producing reports concerning its state of progress, verifying that the e-CRFs are updated (request for additional information, corrections, etc.), and transmitting information on severe AEs to the sponsor. The assistant will follow the SOPs. A data safety monitoring board (DSMB) will be composed of three medical doctors specialized in pharmaco-dermatology and dermatology. The DSMB will be systematically contacted: (1) at any time by the sponsor for each case of expected serious adverse reaction or for a suspected unexpected serious adverse drug reaction (SUSAR); (2) before each development safety update report is sent to the French Agency for the Safety of Health Products (ANSM); and (3) if data may change the benefit/risk ratio during the clinical trial. The study may be stopped definitely or temporarily at any time by the sponsor on the basis of information provided by the DSMB. The DSMB will meet in case of a SUSAR, new fact, new safety information that could lead to re-evaluation of the benefit/risk ratio of the participant, and new scientific information challenging study continuation.

The sponsor reserves the right to interrupt definitively the study at any time if it appears that the inclusion objectives have not been met.

There is no prespecified interim analysis and no statistical stopping rules.

### Harms

All AEs will be monitored until they are completely resolved. The investigator will immediately notify the sponsor of any serious AE. The sponsor will report all SUSARs to the Eudravigilance (European pharmacovigilance database), French health authorities (ANSM), and the investigators within the regulatory time periods for reporting.

### Auditing

An audit may be performed at any time by sponsor-appointed individuals who are independent of those responsible for the study. The investigators agree to comply with the requirements of the sponsor and the relevant authority for an audit or inspection of the study. The audit can apply at all stages of the study, from development of the protocol to publication of results.

### Ethics and dissemination

#### Research ethics approval

The sponsor and the investigators undertake to conduct this study in compliance with French law no. 2004–806 of 9 August 2004 and following Good Clinical Practice and the Helsinki Declaration (Ethical Principles for Medical Research involving Human Subjects, Tokyo 2004). The study will be conducted in accordance with this protocol. With the exclusion of emergency situations requiring specific therapeutic actions, the investigators will observe the protocol in all respects, particularly in obtaining consent and the notification and follow-up of serious AEs.

The protocol was approved by the French institutional review board (18.07.19.74306) and received authorization from ANSM.

### Protocol amendments

Important protocol modifications will be submitted for approval to the institutional review board of the University Hospital of Tours and will be communicated to coinvestigators.

### Consent and assent

Participants will be orally informed of the study and will receive written information; their informed sign consent will be obtained (Additional file 1). For children aged < 18 years, parents will give their informed signed consent after their child has consented (if able). Children aged ≥ 16 years must also consent to use of their data according to article 89 (Regulation [EU] 2016/679 - RGPD). No biological specimens for genetic or molecular analysis will be collected.

### Confidentiality

During this biomedical research study or when it is completed, the information collected for participants and forwarded to the sponsor by the investigators (or any other specialized staff member involved) will be made anonymous. Under no circumstances will the uncoded names or addresses of the participants concerned appear in any data.

### Access to data

The sponsor is responsible for obtaining agreement from all parties involved in the study in order to guarantee direct access (in all sites where the study is being conducted) to source data, source documents, and reports, to control their quality, and to audit them.

The investigators will make available to people with right of access according to the legislative and regulatory provisions in force (articles L.1121–3 and R.5121–13 of the French Public Health Act) the documents and individual data strictly necessary for monitoring, performing quality control, and auditing the biomedical research.

### Ancillary and post-trial care

The current trial is not planned to include patient ancillary care or post-trial care. On completion of the trial, topical sirolimus could be continued and will be on the investigator’s initiative.

### Dissemination policy

INSERM CIC 1415 Tours will analyze the data provided by the study centers. Results will be displayed in a written report that will be submitted to the sponsor. At the end of the analysis, results will be published at ClinicalTrials.gov. The international rules for writing and publication (Vancouver Agreement, February 2006) will be followed. The study results will be presented at scientific conferences and in peer-reviewed publication. We do not intend to use a professional writer. All study investigators will be eligible for authorship depending on contributions to the trial, analysis, and manuscript preparation.

Patients will be informed, at their request, about the overall results of the study.

### SPIRIT

This protocol has been written in accordance with the Standard Protocol Items: Recommendations for Interventional Trials (SPIRIT) guidelines. The SPIRIT checklist is in Additional file 2.

## Discussion

This within-person, blinded RCT aims to compare 0.1% topical sirolimus with topical vehicle in CMLMs in children aged > 6 years and adults. Several case reports have suggested efficacy of this treatment, linked to the anti-lymphangiogenic properties of sirolimus, but this needs to be demonstrated. CMLMs are very rare diseases and this design allows for reducing the number of patients to be included; indeed, the population is too rare for a parallel-group trial. Furthermore, the location and severity of CMLMs are heterogeneous among patients; a within-person design, also called a split-body design, has the advantage of reducing individual inter-variability. Finally, all patients will receive the experimental treatment, which would not be the case with a parallel-group trial.

The hypothesis of this protocol study is that 0.1% topical sirolimus applied once daily during 12 weeks on CMLM is more efficient than a placebo cream (control), by reducing the thickness of CMLM, oozing, bleeding, impairments, and pain. In the literature, modalities of use of topical sirolimus are highly heterogeneous: the product has been used as a cream or solution, with concentrations in the range of 0.015%–8%. Creams were better tolerated than solutions; the most frequently used was sirolimus cream 0.1%, all conditions included [28]. This heterogeneity is linked to the fact that topical sirolimus is not marketed and therefore different formulations are used. Sirolimus has high molecular weight, 914.17 Da [29], which allows for diffusion limited to the skin. Thus, rapamycin was solubilized in a solvent (Transcutol®), which is an excellent permeation agent that enhances drug diffusion through the skin [30].

We expect no general side effects and no severe local adverse effects: for topical sirolimus, adverse effects previously described are local and of mild intensity (irritation, burning sensation, pruritus), reported in one-third of cases. We also expect a non-significant blood passage of the drug. Indeed, in previous reports, systemic passage of sirolimus with different concentrations of topical sirolimus in different skin conditions was not, or almost not, detectable and depended on the quantity of active principle administered and the surface to be treated [28]. In the present study, if systemic passage of sirolimus is evidenced, the consequence would be a reduced difference between the two areas; this would be unfavorable to our expected results.

Current treatments in CMLM are often disappointing and painful (lasers, surgery, sclerotherapy), so we expect an individual benefit with sirolimus. If the trial demonstrates the efficacy and safety of the treatment in patients with CMLMs, this will lead to a real change in the management of this rare condition and 0.1% sirolimus cream would become the first-line treatment.

## Trial status

The current version of the protocol is V1.3, dated 21 December 2018.

This study was due to start recruitment in June 2019 and have a total period of recruitment of 24 months (June 2021).

## Supplementary information

**Additional file 1.** Informed consent.

**Additional file 2.** SPIRIT checklist.

## Data Availability

The datasets used and/or analyzed during the current study are available from the corresponding author on reasonable request.

## Abbreviations

AEs: Adverse events
ANSM: Agence Nationale de Sécurité du Médicament et des produits de santé
CMLM: Cutaneous microcystic lymphatic malformation
DLQI: Dermatology Life Quality Index
DSMB: Data Safety Monitoring Board
e-CRF: Electronic case report form
FTU: Fingertip unit
mTOR: Mammalian target of rapamycin
PGA: Physician Global Assessment
RCT: Randomized controlled trial
SOP: Standardized Operating Procedures
SUSAR: Suspected unexpected serious adverse reaction
VAS: Visual analog scale
VM: Vascular malformation
