# Supplementary material for: Topical sirolimus 0.1% for treating cutaneous microcystic lymphatic malformations in children and adults (TOPICAL): protocol for a multicenter phase 2, within-person, randomized, double-blind, vehicle-controlled clinical trial
Source: Trials. 2019 Dec 17;20:739. doi: 10.1186/s13063-019-3767-8 (PMC6918625; doi:10.1186/s13063-019-3767-8)
Supplement: Supplementary file 1 — Additional file 1. Informed consent. [file 13063_2019_3767_MOESM1_ESM.pdf]

Formulaire de consentement de participation à une Recherche interventionnelle sur la personne humaine : **Mineurs participant à l'étude**

Parent (1) : Je soussigné(e),
Nom : ………………………. *Prénom :* ……………………………......
Adresse : ……………………………………………………………….
…………………………………………………………………………….

Parent (2) : Je soussigné(e),
Nom : ………………………. *Prénom :* ……………………………......
Adresse (à compléter si différente de (1)) : ……….………
…………………………………………………………………………….

Merci de cocher seulement si applicable : ☐ Certifie être le seul titulaire de l'exercice de l'autorité parentale

De l'enfant *Nom :* ………………………………. *Prénom :* ……………………………….. **Né(e) le :** ___ / ___ / _______

Déclarent avoir pris connaissance des informations orales et écrites (*lettres d'information v1-2-PARENTS du 21/12/2018*) qui nous ont été transmises pour une **participation de notre enfant à l'étude clinique TOPICAL** « *Évaluation de l'efficacité et de la tolérance du sirolimus topique 0.1% dans les malformations cutanées lymphatiques microkystiques : essai thérapeutique de phase II, randomisé, en double aveugle, en comparaison intra individuelle versus véhicule* » organisée par le CHRU de Tours qui en est le Promoteur, autorisée par l'ANSM (n° MEDAECNAT-2018-12-00055) et le CPP Nord-Ouest 1 (n° 18.07.19.74306).

Nous avons reçu du médecin investigateur qui suit notre enfant, toutes les informations nécessaires concernant l'objectif de cette recherche, son déroulement ainsi que les conditions de participation de notre enfant, nos droits, les bénéfices attendus, les contraintes et les risques prévisibles. Nous avons pu poser toutes les questions que nous voulions et nous avons reçu les réponses adaptées. Nous avons bénéficié d'un temps de réflexion suffisant entre ces informations et le présent consentement. Nous avons également compris que nous pouvions refuser que notre enfant participe à cette étude sans que cela ne porte préjudice à la suite de sa prise en charge.

Compte tenu des informations qui nous ont été transmises,

- **Nous acceptons librement et volontairement que notre enfant participe à cette étude,**

- **Nous acceptons que les données enregistrées à l'occasion de cette étude puissent faire l'objet d'un traitement informatisé par le promoteur ou pour son compte dans les conditions prévues par la Loi Informatique et Libertés, de la loi n° 2018-493 du 20 juin 2018 relative à la protection des données personnelles et du Règlement (UE) 2016/679 du Parlement européen et du Conseil du 27 avril 2016. Nous avons été informés de tous nos droits, ainsi que des droits de notre enfant, concernant ces données personnelles selon les modalités décrites dans la lettre d'information en vigueur.** Nous avons été informés que notre enfant doit également donner son consentement pour le traitement informatisé de ses données, et qu'il peut y consentir seul à compter de l'âge de quinze ans. Si notre enfant a quinze ans, ou plus, il a également le droit de s'opposer à ce que nous ayons accès ou soyons informés du traitement de ses données au cours de la recherche.

- **Nous certifions être bénéficiaires d'un régime de sécurité sociale.**

Pour les jeunes filles réglées : une contraception efficace devra être utilisée jusqu'à la fin de l'étude.

Conformément à la loi, le CHRU de Tours, promoteur de l'étude, a souscrit une assurance de responsabilité auprès de la compagnie SHAM afin de couvrir tout préjudice corporel ou toute incapacité que pourrait entraîner cette étude.

Partie à remplir par les parents (ou titulaire de l'exercice de l'autorité parentale)	
Date : __ / __ / ____ Signature :	Date : __ / __ / ____ Signature :

Partie à remplir par le participant mineur
J'ai bien été informé(e) de l'étude et j'ai reçu une lettre d'information. J'accepte le traitement informatisé de mes données.<br>Nom, Prénom : ……………………………………………………………………. Date : __ / __ / ____ Signature :

Partie à remplir par le médecin investigateur
Nom, Prénom : ……………………………………………………………………. Date : __ / __ / ____ Signature :<br>*Merci de cocher seulement si applicable :* ☐ Enfant dans l'incapacité de donner son consentement ci-dessus.

En trois exemplaires : original pour le médecin investigateur, un exemplaire pour les parents, et un exemplaire pour le promoteur

Formulaire de consentement de participation à une Recherche interventionnelle sur la personne humaine : **Participants devenus majeurs en cours d'étude**

Je soussigné(e) *Nom :* ………………………………………… ***Prénom :*** …………………………………………

Adresse : ………

………

Déclare avoir pris connaissance des informations orales et écrites (*lettre d'information v1-MAJEUR du 21/12/2018*) qui m'ont été transmises afin de confirmer ma **participation à l'étude clinique TOPICAL** « *Évaluation de l'efficacité et de la tolérance du sirolimus topique 0.1% dans les malformations cutanées lymphatiques microkystiques : essai thérapeutique de phase II, randomisé, en double aveugle, en comparaison intra individuelle versus véhicule* » organisée par le CHRU de Tours qui en est le Promoteur, autorisée par l'ANSM (n° MEDAECNAT-2018-12-00055) et le CPP Nord-Ouest 1 (n° 18.07.19.74306).

Je confirme voir reçu du médecin investigateur, toutes les informations nécessaires concernant l'objectif de cette recherche, son déroulement ainsi que mes conditions de participation, mes droits, les bénéfices attendus, les contraintes et les risques prévisibles. J'ai pu poser toutes les questions que je voulais et j'ai reçu les réponses adaptées. J'ai bénéficié d'un temps de réflexion suffisant entre ces informations et le présent consentement. J'ai également compris que je pouvais refuser de continuer de participer à cette étude sans que cela ne porte préjudice à la suite de ma prise en charge. Compte tenu des informations qui m'ont été transmises,

- J'accepte librement et volontairement de continuer de participer à cette étude,

- J'accepte que les données enregistrées à l'occasion de cette étude puissent faire l'objet d'un traitement informatisé par le promoteur ou pour son compte dans les conditions prévues par la Loi Informatique et Libertés, de la loi n° 2018-493 du 20 juin 2018 relative à la protection des données personnelles et du Règlement (UE) 2016/679 du Parlement européen et du Conseil du 27 avril 2016. J'ai été informé(e) de tous mes droits concernant mes données personnelles selon les modalités décrites dans la lettre d'information en vigueur,

- Je certifie être bénéficiaire d'un régime de sécurité sociale.

- Si je suis une jeune femme, je m'engage à utiliser une méthode de contraception efficace jusqu'à la fin de l'étude.

Conformément à la loi, le CHRU de Tours, promoteur de cette étude, a souscrit une assurance de responsabilité auprès de la compagnie SHAM afin de couvrir tout préjudice corporel ou toute incapacité que pourrait entraîner cette étude.

Partie à remplir par le participant à l'étude	***Partie à remplir par le médecin investigateur***
Nom et prénom :<br>_______________________________________	Nom et prénom :<br>_______________________________________
Date : ____ / ____ / ________<br>Signature :	Date : ____ / ____ / ________<br>Signature :

En trois exemplaires : original pour le médecin investigateur, un exemplaire pour le participant, et un exemplaire pour le promoteur

Formulaire de consentement de participation à une Recherche interventionnelle sur la personne humaine : **Adultes participant à l'étude**

Je soussigné(e) ***Nom :*** ……………………………………… ***Prénom :*** ………………………………………

Adresse : ……

……

Déclare avoir pris connaissance des informations orales et écrites (*lettre d'information v1-ADULTE du 21/12/2018*) qui m'ont été transmises pour une **participation à l'étude clinique TOPICAL** « *Évaluation de l'efficacité et de la tolérance du sirolimus topique 0.1% dans les malformations cutanées lymphatiques microkystiques : essai thérapeutique de phase II, randomisé, en double aveugle, en comparaison intra individuelle versus véhicule* » organisée par le CHRU de Tours qui en est le Promoteur, autorisée par l'ANSM (n° MEDAECNAT-2018-12-00055) et le CPP Nord-Ouest 1 (n° 18.07.19.74306).

J'ai reçu du médecin investigateur, toutes les informations nécessaires concernant l'objectif de cette recherche, son déroulement ainsi que mes conditions de participation, mes droits, les bénéfices attendus, les contraintes et les risques prévisibles. J'ai pu poser toutes les questions que je voulais et j'ai reçu les réponses adaptées. J'ai bénéficié d'un temps de réflexion suffisant entre ces informations et le présent consentement. J'ai également compris que je pouvais refuser de participer à cette étude sans que cela ne porte préjudice à la suite de ma prise en charge. Compte tenu des informations qui m'ont été transmises,

- J'accepte librement et volontairement de participer à cette étude,

- J'accepte que les données enregistrées à l'occasion de cette étude puissent faire l'objet d'un traitement informatisé par le promoteur ou pour son compte dans les conditions prévues par la Loi Informatique et Libertés, de la loi n° 2018-493 du 20 juin 2018 relative à la protection des données personnelles et du Règlement (UE) 2016/679 du Parlement européen et du Conseil du 27 avril 2016. J'ai été informé(e) de tous mes droits concernant mes données personnelles selon les modalités décrites dans la lettre d'information en vigueur,

- Je certifie être bénéficiaire d'un régime de sécurité sociale.

- Si je suis une femme en âge de procréer, je m'engage à utiliser une méthode de contraception efficace jusqu'à la fin de l'étude.

Conformément à la loi, le CHRU de Tours, promoteur de cette étude, a souscrit une assurance de responsabilité auprès de la compagnie SHAM afin de couvrir tout préjudice corporel ou toute incapacité que pourrait entraîner cette étude.

Partie à remplir par le participant à l'étude	***Partie à remplir par le médecin investigateur***
Nom et prénom :<br>______________________________	Nom et prénom :<br>______________________________
Date : ____ / ____ / ________<br>Signature :	Date : ____ / ____ / ________<br>Signature :

En trois exemplaires : original pour le médecin investigateur, un exemplaire pour le participant, et un exemplaire pour le promoteur
